# Adverse Prognostic Impact of the *KIT* D816V Transcriptional Activity in Advanced Systemic Mastocytosis

**DOI:** 10.3390/ijms22052562

**Published:** 2021-03-04

**Authors:** Nicole Naumann, Johannes Lübke, Sofie Baumann, Juliana Schwaab, Oliver Hoffmann, Sebastian Kreil, Vito Dangelo, Lukas Reiter, Peter Bugert, Thomas Kristensen, Karl Sotlar, Verena Haselmann, Sven Schneider, Georgia Metzgeroth, Christel Weiss, Henning D. Popp, Alice Fabarius, Wolf-Karsten Hofmann, Nicholas C. P. Cross, Andreas Reiter, Mohamad Jawhar

**Affiliations:** 1 Department of Hematology and Oncology, University Hospital Mannheim, Heidelberg University, 68167 Mannheim, Germany; nicole.naumann@medma.uni-heidelberg.de (N.N.); johannes.luebke@medma.uni-heidelberg.de (J.L.); sofie.baumann@medma.uni-heidelberg.de (S.B.); juliana.schwaab@medma.uni-heidelberg.de (J.S.); oliver.hoffmann@medma.uni-heidelberg.de (O.H.); sebastian.kreil@medma.uni-heidelberg.de (S.K.); vito.dangelo@medma.uni-heidelberg.de (V.D.); hi224@stud.uni-heidelberg.de (L.R.); g.metzgeroth@medma.uni-heidelberg.de (G.M.); henning.popp@medma.uni-heidelberg.de (H.D.P.); alice.fabarius@medma.uni-heidelberg.de (A.F.); w.k.hofmann@medma.uni-heidelberg.de (W.-K.H.); andreas.reiter@medma.uni-heidelberg.de (A.R.); 2 Institute of Transfusion Medicine and Immunology, University Hospital Mannheim, Heidelberg University, 68167 Mannheim, Germany; peter.bugert@medma.uni-heidelberg.de; 3 Mastocytosis Center, Odense University Hospital, 5000 Odense, Denmark; thomas.kielsgaard.kristensen@rsyd.dk; 4 Department of Pathology, Odense University Hospital, 5000 Odense, Denmark; 5 University Hospital Salzburg, Paracelsus Medical University, 5020 Salzburg, Austria; k.sotlar@salk.at; 6 Institute for Clinical Chemistry, University Hospital Mannheim, Heidelberg University, 68167 Mannheim, Germany; verena.haselmann@umm.de (V.H.); sven.schneider@klinikum-passau.de (S.S.); 7 Department of Medical Statistics and Biomathematics, University Hospital Mannheim, Heidelberg University, 68167 Mannheim, Germany; christel.weiss@medma.uni-heidelberg.de; 8 School of Medicine, University of Southampton, Southampton SO17 1BJ, UK; ncpc@soton.ac.uk; 9 Wessex Regional Genetics Laboratory, Salisbury SP2 8BJ, UK

**Keywords:** advanced SM, *KIT* D816V, allele burden, variant allele frequency, survival

## Abstract

In systemic mastocytosis (SM), qualitative and serial quantitative assessment of the *KIT* D816V mutation is of diagnostic and prognostic relevance. We investigated peripheral blood and bone marrow samples of 161 patients (indolent SM (ISM), *n* = 40; advanced SM, AdvSM, *n* = 121) at referral and during follow-up for the *KIT* D816V variant allele frequency (VAF) at the DNA-level and the *KIT* D816V expressed allele burden (EAB) at the RNA-level. A round robin test with four participating laboratories revealed an excellent correlation (*r* > 0.99, *R^2^* > 0.98) between three different DNA-assays. VAF and EAB strongly correlated in ISM (*r =* 0.91, coefficient of determination, *R^2^ =* 0.84) but only to a lesser extent in AdvSM (*r =* 0.71; *R^2^* = 0.5). However, as compared to an EAB/VAF ratio ≤2 (cohort A, 77/121 patients, 64%) receiver operating characteristic (ROC) analysis identified an EAB/VAF ratio of >2 (cohort B, 44/121 patients, 36%) as predictive for an advanced phenotype and a significantly inferior median survival (3.3 vs. 11.7 years; *p* = 0.005). In terms of overall survival, Cox-regression analysis was only significant for the EAB/VAF ratio >2 (*p* = 0.006) but not for VAF or EAB individually. This study demonstrates for the first time that the transcriptional activity of *KIT* D816V may play an important role in the pathophysiology of SM.

## 1. Introduction

Systemic mastocytosis (SM) is a rare hematologic neoplasm characterized by clonal expansion and abnormal accumulation of neoplastic mast cells in various organ systems. According to the World Health Organization (WHO), SM can be divided into indolent SM (ISM) and advanced SM (AdvSM), which is further subcategorized into aggressive SM (ASM), SM with associated hematologic neoplasm (SM-AHN) and mast cell leukemia (MCL) [1,2,3]. ISM patients have a nearly normal life expectancy while AdvSM patients have a poor survival of median three to four years [4,5,6,7].

*KIT* D816V is the pathogenic driver mutation and is detectable in more than 90% of SM patients. Qualitative detection of *KIT* D816V has been established as a diagnostic criterion for SM. The serial quantitative assessment of the *KIT* D816V expressed allele burden (EAB) by a real time RT-quantitative PCR (RT-qPCR) assay during treatment with the KIT-inhibitor midostaurin is a strong and independent marker for response, progression and survival [8,9].

DNA-based quantitative assays (variant allele frequency, VAF) are more widely used than RNA-based assays [10,11], but only limited data exist concerning the reproducibility between different assays and the correlation between the DNA- and RNA-based quantitative assays [12,13,14,15,16]. We, thus, sought to quantitatively assess *KIT* D816V at both the DNA- and RNA-levels in bone marrow (BM) and peripheral blood (PB) samples obtained at referral and during follow-up from patients with ISM and AdvSM.

## 2. Results

### 2.1. Patients’ Characteristics 

Patients’ characteristics are listed in Table 1. The subcategories of AdvSM included ASM (18/121, 15%), MCL (2/121, 2%) and SM/MCL-AHN (101/121, 83%). Eighteen AdvSM patients (18/121, 16%) had progression to secondary acute myeloid leukemia (AML) (11/19, 61%) or secondary MCL (7/19, 39%). Fifty-seven AdvSM patients (47%) were treated with the multikinase/KIT-inhibitor midostaurin. Significant differences between ISM (*n* = 40) and AdvSM (*n* = 121) included gender (female 43%, male 67%, *p* = 0.006), age (median 54 vs. 76 years, *p* < 0.0001), hemoglobin (median 13.9 g/dL versus 10.8 g/dL, *p* < 0.0001), platelets (median 283 × 10^9^/L vs. 114 × 10^9^/L, *p* = 0.0002), serum tryptase level (median 46 µg/L versus 180 µg/L, *p* < 0.0001), alkaline phosphatase (median 76 U/L vs. 200 U/L), and overall survival (OS, median not reached vs. 4.8 years, *p* < 0.0001).

### 2.2. Assessment of Analytical Sensitivity, Specificity and Reproducibility of the dPCR Assay

**Figure 1:**
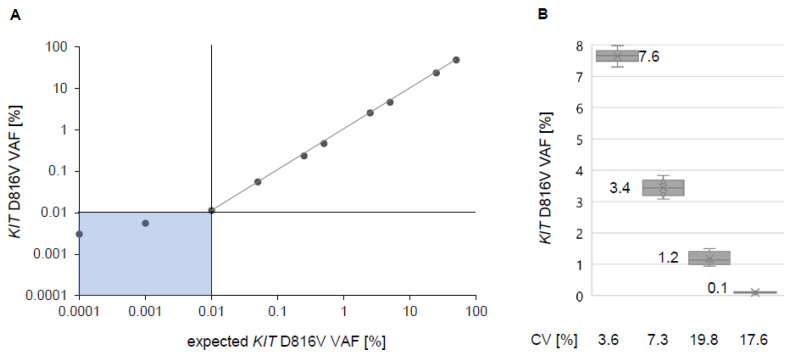
Limit of detection (LOD) and reproducibility of digital PCR (dPCR) for quantitative assessment of the *KIT* D816V variant allele fraction (VAF). (**A**) dPCR of a dilution series of a patient sample with 50% VAF. Single points represent merged measurements from multiple chips (*n* = 3). Dilution results are linear down to 0.1% VAF. (**B**) Reproducibility of four patient samples from 0.1 to 7.6% *KIT* D816V VAF (measured with at least 3 replicates) showing a coefficient of variation (CV) below 20% for all samples.

For evaluation of LOD, we performed a serial dilution series with DNA isolated from a PB sample with a heterozygous mutation status and a VAF of 50% ± 0.3% (mean ± standard deviation). On average, the total number of wildtype *KIT* transcripts per dPCR reaction ranged from 50,000 to 100,000 molecules. If exactly one *KIT* D816V transcript is detectable in a single PCR reaction, a VAF of 0.001% is theoretically achievable. Based on a strong linear correlation of *r =* 0.99, our serial dilution series showed in practice a LOD of 0.01% on average (Figure 1A). For a mathematical definition of the LOD, we determined the LOB. Up to two *KIT* D816V positive events were measured in *n* = 6 healthy individuals. Therefore, LOB was defined as 0.0025%. Finally, the replicate measurement of three low-level positive samples (mean <0.06% VAF) allowed assigning the LOD of 0.04%. A sample was assessed as positive upon the presence of at least three *KIT* D816V signals per measurement.

For validation of reproducibility, we performed LOQ experiments on four samples with low, and high VAF (0.1% to 7.6%), respectively. As a quantity for LOQ, we determined the coefficient of variation (CV) for all samples with values between 3.6% for the highest VAF and 17.6% for the lowest VAF (three samples measured in five independent experiments), which is consistent with that reported for quantitative PCR (Figure 1B) [17,18].

### 2.3. Inter-Laboratory Round-Robin Test

**Figure 2:**
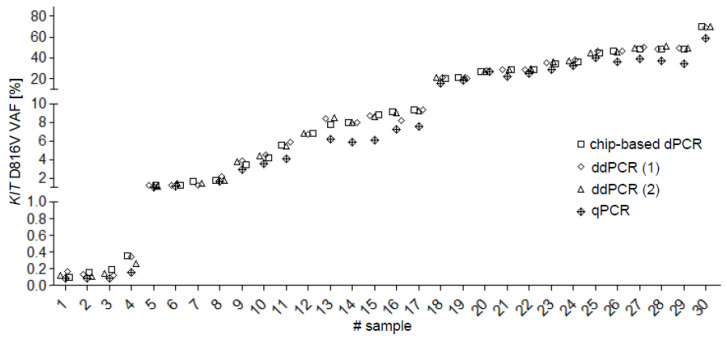
Quantitative assessment of the *KIT* D816V variant allele fraction (VAF) in 30 samples using various PCR methods. A very good correlation was observed for dPCR vs. ddPCR (*r =* 0.99, *R^2^ =* 0.99) and for dPCR vs. qPCR (*r =* 0.99, *R^2^ =* 0.98). dPCR, digital PCR; (1) ddPCR, droplet digital PCR from laboratory A; (2) ddPCR droplet digital PCR from laboratory B; qPCR, genomic quantitative real-time PCR.

In the inter-laboratory round-robin test (labs, *n* = 4; samples, *n* = 30), an excellent correlation was observed between the different DNA-based assays (dPCR versus ddPCR: *R^2^ =* 0.99; dPCR vs. qPCR: *R^2^ =* 0.98) (Figure 2).

### 2.4. Comparison of VAF between PB and BM

**Figure A1:**
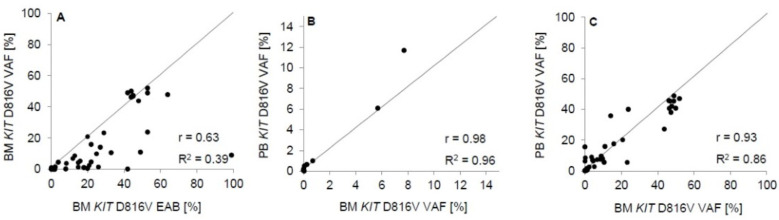
Comparison of VAF and/or EAB in PB and/or BM. (**A**) The comparison of VAF vs. EAB in BM form AdvSM patients (*n* = 40) revealed a correlation of *r* = 0.63 (*R^2^* = 0.39). The comparison between the VAF in PB and BM revealed a correlation of *r* = 0.98 (*R ^2^*= 0.96) in ISM (*n* = 8) (**B**) and *r* = 0.93 (*R^2^* = 0.86) in AdvSM (*n* = 37) (**C**); VAF, variant allele fraction; EAB, expressed allele burden; PB, peripheral blood; BM, bone marrow.

The comparison between the VAF in PB and BM revealed a correlation of *r =* 0.98 (*R^2^ =* 0.96) in ISM (*n* = 8) and *r =* 0.93 (*R^2^ =* 0.86) in AdvSM (*n* = 37), respectively (Appendix A Figure A1).

### 2.5. Comparison between EAB and VAF

**Figure 3:**
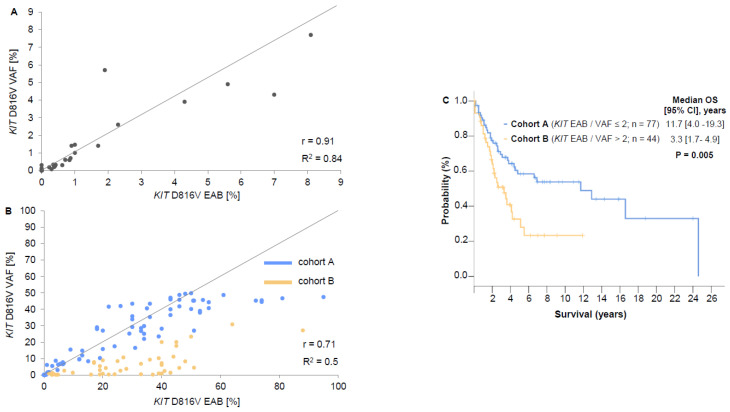
Comparison between expressed allele burden (EAB, RNA/cDNA) and variant allele fraction (VAF, gDNA) in indolent systemic mastocytosis (ISM, *n* = 40) and advanced SM (AdvSM, *n* = 121). The correlation between EAB and VAF showed a strong linear relationship in ISM patients (**A**) but only to a lesser extent in AdvSM patients (**B**). Cohort A represents patients with an EAB/VAF ration ≤ 2 (blue) while cohort B represents patients with an EAB/VAF ratio > 2 (yellow). (**C**) The overall survival (OS) of cohort B (*p* = 0.005). In nine patients *KIT* D816V was below 1 % at cDNA and DNA level. Independent of their ratio they were categorized as “no significant change” (ratio ≤ 2, blue, cohort A).

In PB of ISM patients (*n* = 40), EAB and VAF had a correlation of *r =* 0.91 (*R^2^ =* 0.84) (Figure 3A). In AdvSM patients, *r* and *R^2^* were significantly inferior (PB, *n* = 121: *r =* 0.71, *R^2^ =* 0.5; BM, *n* = 37: *r =* 0.63; *R^2^ =* 0.39). ROC analysis showed an ideal threshold for an EAB/VAF ratio of 2 for cohort classification. In PB, the EAB/VAF ratio was ≤2 (cohort A) in 77/121 (64%) and ≥2 (cohort B) 44/121 (36%) of AdvSM patients (Figure 3B, Appendix A Figure A1A).

**Figure 4:**
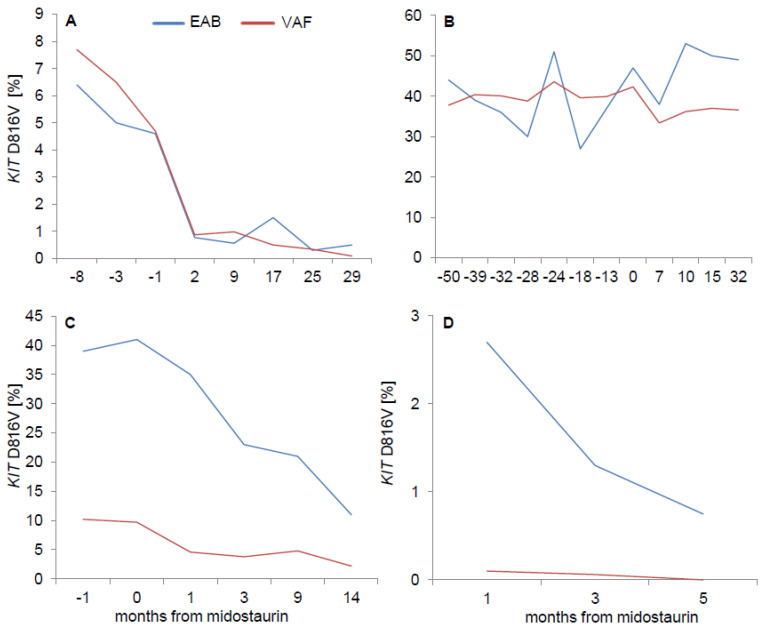
Serial measurement of expressed allele burden (EAB, cDNA) and variant allele fraction (VAF, gDNA) on midostaurin. Irrespective of the cohorts (cohort A: EAB/VAF ≤ 2, (**A**,**B**); cohort B: EAB/VAF > 2, (**C**,**D**)), the changes of *KIT* EAB and VAF nearly paralleled each other.

To confirm the significant disparity between EAB and VAF in individual patients of cohort B, contemporaneously obtained BM and PB from 12 patients were investigated. In the vast majority of patients (9/12, 75%), the EAB/VAF ratio of >2 could be confirmed in BM, while it was between 1 and 2 in 3/12 (25%) patients. Serial/longitudinal analyses of at least three PB samples in 12 patients revealed a stable EAB/VAF ratio during follow-up. Out of these, eight AdvSM patients were serially investigated while on treatment with the multikinase/KIT-inhibitor midostaurin. *KIT* EAB and VAF paralleled each other throughout the follow-up (Figure 4).

### 2.6. Disease Characteristics in Cohorts A and B

Significant differences between cohorts A and B were observed in terms of a higher median hemoglobin level (*p* = 0.006), a lower percentage of patients with hemoglobin <10g/dL (*p* = 0.01), a lower median monocyte level (*p* = 0.01), a lower percentage of patients with alkaline phosphatase level >150 U/L (*p* = 0.01), a lower number of patients with a high risk molecular profile (at least one gene mutation in *SRSF2*, *ASXL1*, and/or *RUNX1*, S/A/R, *p* = 0.02) and a lower median vitamin B12 level (*p* = 0.02) in cohort A (Table 2). Patients of cohort A had a significantly better OS than patients in cohort B (median OS 11.7 versus 3.3 years; hazard ratio (HR) 2.1; 95% confidence interval (95%CI) 1.2–3.6; *p* = 0.005) (Figure 3C).

### 2.7. Prognostic Value of EAB, VAF and EAB/VAF Ratio

In terms of OS, Cox-regression analysis was only significant for the EAB/VAF ratio >2 (*p* = 0.006) but not for VAF (*p* = 0.657) or (EAB = 0.658) individually.

## 3. Discussion

In the vast majority of patients with ISM, the *KIT* D816V variant allele fraction (VAF) is rather low, e.g., less than 1–3% in 35/40 (88%) samples. In these cases, the sensitivity of qPCR or dPCR (both <0.01%) for detection of low level *KIT* D816V mutation is superior to next-generation sequencing (NGS, sensitivity >1–3%) and NGS may even fail to identify the *KIT* D816V mutation. We consider NGS as the appropriate tool for identification of additional somatic mutations [4,6]. While BM MC infiltration and serum tryptase represent the *KIT* D816V positive mast cell burden, the *KIT* D816V VAF/EAB reveals the overall disease burden including the involvement of non-mast lineages, e.g., neutrophils, monocytes and eosinophils. This so-called multilineage involvement is identified in 60–80% of patients with AdvSM. In SM-AHN, the frequently observed discrepancy between a high *KIT* D816V VAF/EAB and a low serum tryptase may indicate a dominant AHN clone. Overall, the median *KIT* D816V VAF/EAB in PB of AdvSM patients is approximately 20–30% and it was recently shown that response monitoring at the molecular level is not only feasible but also highly informative [4,10,11,14,16].The reduction of the *KIT* D816V EAB >25% at month 6 is the most favorable predictor for improved survival in midostaurin-treated AdvSM patients [8]. In consequence of the increased diagnostic and prognostic relevance of quantitative PCR assays for *KIT* D816V, we evaluate the comparability of various DNA assays and compare DNA-based dPCR with qPCR at RNA/cDNA level.

While real-time PCR (qPCR) utilizes the absolute quantification of a somatic mutation relative to a calibrator, dPCR is a method for the absolute quantification of a target in the absence of a calibrator. Several dPCR platforms have recently been developed but data from round-robin testing as an external quality assessment has been lacking. We, thus, performed an international inter-laboratory comparison of four laboratories upon quantification of the *KIT* D816V VAF by chip-based dPCR, ddPCR (droplets of an emulsion for partition of PCR reactions) and qPCR which revealed an excellent correlation (*r =* 0.99, *R^2^ =* 0.99) in samples derived from patients with ISM and AdvSM. dPCR offers a reliable and reproducible tool for quantification of *KIT* D816V and should be considered as candidate for inter-laboratory standardization and regular use for diagnosis and response monitoring in clinical trials and daily routine.

Although sensitivity and specificity are comparable, only limited data exist upon the comparability between *KIT* D816V VAF and EAB [10,14,19]. We investigated a large cohort of patients with ISM and AdVSM unveiling an excellent correlation in ISM but not in AdvSM. In more detailed analyses, two different AdvSM cohorts were identified in which approximately two-thirds of patients had an excellent correlation comparable to ISM whereas in approximately one-third of patients the *KIT* D816V EAB was at least 2-fold higher than the VAF, suggesting increased transcriptional activity of *KIT* D816V relative to the size of the mutant clone. We confirmed this significant disparity between EAB and VAF by finding; (i) identical results by dPCR and ddPCR in two independent laboratories in the majority of patients, (ii) comparable EAB/VAF ratios in contemporaneously obtained samples from BM and PB in the vast majority of patients; and (iii) comparable EAB/VAF ratios in serial analyses of at least 3 PB samples in the same individual.

In terms of OS, Cox-regression analysis was only significant for the EAB/VAF ratio >2 (*p* = 0.006) but not for VAF or EAB individually, highlighting a *KIT* D816V EAB/VAF ratio ≥ 2 at diagnosis as an adverse prognostic marker for OS in AdvSM. Patients with an EAB/VAF ratio >2 had a more advanced phenotype (e.g., lower hemoglobin level, higher monocytes level, higher alkaline phosphatase level, higher number of high-risk mutations) and inferior survival. The trigger mechanisms for the supposed enhanced transcriptional activity remain to be determined. To date, there are only a few reports comparing mutational analysis at DNA and RNA/cDNA level in hematological neoplasms. A discrepancy has been reported regarding the *JAK2* V617F mutation in patients with essential thrombocythemia and polycythemia vera, and also regarding the type A mutation of *NPM1* in acute myeloid leukemia (AML) [20,21,22]. All reports found significantly higher mutation levels at RNA/cDNA level compared to DNA-level highlighting the potential superior sensitivity of RNA-based assays and the possible impact of this discrepancy on disease phenotype and prognosis.

In conclusion, (i) dPCR is a sensitive and reliable assay for assessment of the *KIT* D816V VAF, (ii) it could serve as standardized tool for optimized comparability within clinical trials and daily routine, (iii) both, the *KIT* D816V VAF and the EAB can be used for subtyping, treatment monitoring and prognostication, (iv) an increased *KIT* D816V transcriptional activity defined by an EAB/VAF ratio ≥2 is associated with a more aggressive phenotype and adverse outcome.

## 4. Material and Methods

### 4.1. Patients and Samples

PB (*n* = 161; ISM, *n* = 40; AdvSM, *n* = 121) and corresponding BM (*n* = 45, AdvSM, *n* = 37; ISM, *n* = 8) samples were collected from *KIT* D816V positive patients at time of referral. For serial analyses of midostaurin treated patients, we analyzed at least three PB samples from 8 patients. All patients were diagnosed and subtyped according to the 2016 WHO classification and were listed within the ‘German Registry for Disorders of Eosinophils and Mast cells‘. Data collection was compliant with the tenets of the Declaration of Helsinki and was approved by the Institutional Review Board of the Medical Faculty Mannheim, Heidelberg University, Germany. All patients gave written informed consent.

### 4.2. RNA-Based Assessment of KIT D816V

Quantitative assessment of the *KIT* D816V expressed allele burden (EAB) at RNA-level was performed by allele-specific RT-qPCR. Two PCR assays were designed for amplification of total *KIT* transcripts and *KIT* D816V mutated transcripts. *KIT* D816V EAB was calculated as ratio between mutant *KIT* D816V and total *KIT* transcripts. Limit of detection reveals a sensitivity of 0.01–0.1%. PCR was performed using the universal “mastermix” (LightCycler^®^ FastStart PLUS set, Roche Diagnostics, Mannheim, Germany) and specific primer and probes on a LightCycler^®^ instrument 1.5 (Roche Diagnostics, Mannheim, Germany) in a final volume of 20 μL with 2 μL cDNA or plasmid product (500 nm primer; 250 nm probes). Thermocycling conditions were as follows: 95 °C (10 min), 45 cycles: 95 °C (1 s), 60 °C (10 s), and 72 °C (26 s) [13].

### 4.3. DNA-Based Assessment of KIT D816V

#### 4.3.1. Chip-Based Digital PCR.

For quantitative assessment of the *KIT* D816V VAF, a digital PCR (dPCR) assay was established. The analysis was performed using the QuantStudio^TM^ three-dimensional (3D) dPCR System (ThermoFisher Scientific, Waltham, MA, USA). Per sample, a 15 µL reaction was prepared. The volume including 7.1 µL of 10 ng/µL DNA, 7.5 µL of QuantStudio^TM^ 3D Digital PCR Master Mix v2 (ThermoFisher Scientific, Waltham, MA, USA) and 0.4 µL of *KIT* D816V specific Taqman gene expression assay (ID: Hs000000039_rm, ThermoFisher Scientific Waltham, MA, USA). The limit of detection (LOD) was assessed through serial dilution experiments with DNA from healthy individuals and from a SM patient with a *KIT* D816V VAF of approximately 50% measured by chip-based dPCR. All samples were analyzed twice in independent PCR runs. dPCR was performed using the following thermal cycling conditions: 96 °C for 10 min, (56 °C for 2 min, 98 °C for 30 s (×39 cycles)) and 56 °C for 2 min.

#### 4.3.2. Droplet Digital PCR.

Measurements were performed using the QX200 Droplet Digital PCR (ddPCR) System (Bio-Rad, Hercules, CA, USA). Per sample, a 22 µL reaction volume including 6 µL (100ng) DNA, 11 µL of ddPCR Supermix for Probes (no UTP, Bio-Rad, Hercules, CA, USA), 3.3 µl H_2_O and 1.1 µL of *KIT* D816V specific primer/probe mix (Bio-Rad, Hercules, CA, USA) were prepared. Twenty µl from this solution was used for droplet generation in the QX200™ Droplet Generator (Bio-Rad, Hercules, CA, USA) followed by PCR analysis and droplet detection using QX200 Droplet Reader (Bio-Rad, Hercules, CA, USA).

#### 4.3.3. Quantitative Real-Time PCR.

qPCR was performed using the 7900HT Fast Real-Time PCR System (Applied Biosystems, Foster City, CA, USA), as previously described [16].

#### 4.3.4. Round-Robin Test for Various DNA-Based PCR Platforms

Thirty PB samples from 26 patients (ISM, *n* = 7; AdvSM, *n* = 19) were used for interlaboratory correlation (round-robin test, *n* = 4; dPCR, *n* = 1; ddPCR, *n* = 2; qPCR, *n* = 1) of VAF results.

#### 4.3.5. Statistical Analysis

All statistical analyses considered clinical and laboratory parameters obtained at the time of diagnosis/first referral. OS analysis was considered from the date of diagnosis to date of death or last visit. OS probabilities were estimated using the Kaplan-Meier method. Pearson correlation analysis was performed for the correlation between two continuous parameters. *t*-test was used to compare continuous variables and medians of distributions. For the destination of hazard ratios, a cox proportional hazard regression model was used. Receiver operating characteristic (ROC) curve was used to select the optimal cut point to dichotomize the EAB/VAF coefficient. All tests were two-sided, with *p* < 0.05 considered as statistically significant.

For dPCR results, absolute quantification, including Poisson quantification algorithm, were performed using the QuantStudio 3D AnalysisSuite Cloud Software online (Thermo Fisher Scientific, Waltham, MA, USA). For evaluation of the limit of detection (LOD), limit of quantification (LOQ) and the limit of blank (LOB) we used established mathematical calculations [17,23] and performed at least three replicates in independent dPCR runs per sample. GraphPad Prism Software (version 6, GraphPad, La Jolla, CA, USA), Excel (version 2019, Microsoft Corporation, Redmond, WA, USA), SPSS (version 21.0.0, IBM Cooperation, Armonk, NY, USA) and SAS software, release 9.4 (SAS Institute, Cary, NC, USA) were used for statistical analysis.

## Figures and Tables

**Table 1:** Clinical, laboratory, outcome and treatment characteristics of patients with indolent systemic mastocytosis (ISM) and advanced SM (AdvSM).

Variables	ISM	AdvSM	*p*-Value
Number of patients (*n*)	40	121	-
Age in years, median (range)	54 (29–83)	76 (30–90)	<0.0001
Male, *n* (%)	17 (43)	81 (67)	0.006
Hemoglobin, g/dL; median (range)	13.9 (11.7–16.8)	10.8 (5.8–15.8)	<0.0001
Platelets, ×10^9^/L; median (range)	283 (87–461)	114 (12–958)	0.0002
MC-infiltration in BM histology, %	not applicable	30 (0–100)	-
Serum tryptase, µg/L; median (range)	46 (8–166)	180 (11–1382)	<0.0001
Alkaline phosphatase, U/L; median (range)	76 (15–166)	200 (33–1279)	<0.0001
**Diagnosis**			
ASM, *n* (%)	-	18 (15)	-
MCL, *n* (%)	-	2 (2)	-
SM/MCL-AHN, *n* (%)	-	101 (83)	-
**Progression to**			
Secondary AML, *n* (%)	-	11 (61)	-
Secondary MCL, *n* (%)	-	7 (39)	-
**Outcome**			
Follow-up, years, median (range)	5 (0–21)	3 (0–25)	n.s.
Death, *n* (%)	0 (100)	60 (50)	<0.0001
Overall survival, median, years	not reached	4.8	<0.0001
**Treatment**			
Midostaurin, *n* (%)	1 (3)	57 (47)	<0.0001

AHN, associated hematological neoplasm; AML, acute myeloid leukemia; ASM, aggressive systemic mastocytosis; BM, bone marrow; MCL, mast cell leukemia; *n*, number.

**Table 2:** Clinical, laboratory, genetic, and outcome characteristics of 121 advanced systemic mastocytosis (AdvSM) patients stratified by an expressed allele burden/variant allele frequency ratio of ≤2 (cohort A) and > 2 (cohort B).

Variables	*KIT* D816V EAB/VAF Ratio ≤ 2 (Cohort A)	*KIT* D816V EAB/VAF Ratio > 2 (Cohort B)	*p*-Value
Number of patients (*n*)	77	44	-
Age in years, median (range)	71 (30–90)	77 (52–88)	-
Male, *n* (%)	49 (63)	32 (73)	-
**Diagnosis**			
ASM, *n* (%)	14 (18)	4 (11)	-
MCL, *n* (%)	2 (3)	-	-
SM/MCL-AHN, *n* (%)	61 (79)	40 (90)	-
**AHN-subtypes**			
MDS/MPN-u, *n* (%)	18 (30)	13 (33)	-
CMML, *n* (%)	27 (44)	17 (43)	-
MDS, *n* (%)	5 (8)	6 (15)	-
MPN-eo, *n* (%)	1 (2)	-	
AML, *n* (%)	1 (2)	1 (2)	-
CEL, *n* (%)	7 (11)	1 (2)	-
PMF, *n* (%)	2 (3)	2 (5)	-
**Progression to**			
AML, *n* (%)	8 (10)	3 (7)	-
MCL, *n* (%)	6 (8)	2 (5)	-
**C-findings**			
Hemoglobin, g/dL; median (range)	11.4 (5.8–15.8)	9.8 (7.5–14.5)	0.006
<10 g/dL; *n* (%)	20 (29)	21 (53)	0.01
Platelets, ×10^9^/L; median (range)	133 (12–618)	106 (28–958)	n.s.
<100 × 10^9^/L, *n* (%)	31 (44)	19 (48)	n.s.
Alkaline phosphatase, U/L; median (range)	188 (33–1206)	303 (53–1279)	n.s.
>150 U/L, *n* (%)	39 (57)	31 (79)	0.01
Albumin, g/L; median (range)	38 (16–48)	36 (22–48)	n.s.
<34 g/L, *n* (%)	23 (34)	14 (40)	n.s.
Ascites, *n* (*%*)	39 (53)	25 (61)	n.s.
**B-findings**			
MC-infiltration in BM histology, %; median (range)	35 (3–95)	30 (0–100)	n.s.
Serum tryptase, µg/L; median (range)	170 (11–1382)	211 (18–875)	n.s.
>100 µg/L, *n* (%)	51 (73)	28 (74)	n.s.
Splenomegaly, *n* (*%*)	60 (87)	37 (90)	n.s.
Hepatomegaly, *n* (*%*)	33 (52)	28 (72)	0.05
**Additional SM and/or AHN relevant findings**			
Leukocytes, ×10^9^/L; median (range)	10.6 (5.8–79.3)	7.6 (1.0–89.4)	n.s.
Monocytes, %; median (range)	7 (1–46)	11 (1–31)	0.01
Eosinophils, %, median (range)	3 (0–81)	6 (0–66)	n.s.
Vitamin B12, ng/L; median (range)	1188 (114–6000)	2842 (489–6000)	0.02
>180 ng/L, *n* (%)	50 (96)	32 (100)	n.s.
*KIT* D816V EAB in PB, %, median (range)	30 (0–95)	28 (2–88)	n.s.
*KIT* D816V VAF in PB, %, median (range)	27.0 (0.0–49.8)	4.0 (0.1–30.8)	<0.001
GI-infiltration, n (*%*)	30 (41)	19 (43)	n.s.
S/A/R mutation(s) ^a^, *n* (%)	38 (51)	31 (74)	0.02
**Treatment**			
Midostaurin ^b^, *n* (%)	26 (48)	14 (39)	n.s.
Cladribine ^b^, *n* (%)	6 (11)	7 (19)	n.s.
Midostaurin + cladribine ^b^, *n* (%)	22 (41)	15 (36)	n.s.
Response to any treatment ^c^, *n* (%)	10 (30)	9 (45)	n.s.
**Outcome**			
Follow-up, years, median (range)	3.5 (0.0–24.6)	2.2 (0.0–11.9)	-
Death, *n* (%)	33 (43)	27 (61)	-

AHN, associated hematological neoplasm; AML, acute myeloid leukemia; ASM, aggressive systemic mastocytosis; BM, bone marrow; CEL, chronic eosinophilic leukemia; CMML, chronic myelomonocytic leukemia; EAB, expressed allele burden; GI, gastrointestinal; MCL, mast cell leukemia; MDS, myelodysplastic syndromes; MPN, myeloproliferative neoplasms; -u, unclassified; -eo, eosinophila; *n*, number; PB, peripheral blood; PMF, primary myelofibrosis; S/A/R, at least one mutation in the *SRSF2*, *ASXL1*, *RUNX1* gene panel; SM, systemic mastocytosis; VAF, variant allele frequency; ^a^ data available for *n* = 75 patients (cohort A) and *n* = 42 patients (cohort B); ^b^ data available for *n* = 54 (70%) patients (cohort A) and *n* = 36 (82%) patients (cohort B); ^c^, data available for *n* = 34 patients (cohort A) and *n* = 20 patients (cohort B).

## Data Availability

The data that support the findings of this study are available from the corresponding author upon reasonable request.
